# Current status and future perspectives of platelet-derived extracellular vesicles in cancer diagnosis and treatment

**DOI:** 10.1186/s40364-024-00639-0

**Published:** 2024-08-26

**Authors:** Tongtao Zhuang, Shenrong Wang, Xiaoqian Yu, Xiaoyun He, Hongbin Guo, Chunlin Ou

**Affiliations:** 1 Department of Pathology, Xiangya Hospital, Central South University, Changsha, 410008 Hunan China; 2 Department of Oncology, Xiangya Hospital, Central South University, Changsha, 410008 Hunan China; 3 Departments of Ultrasound Imaging, Xiangya Hospital, Central South University, Changsha, 410008 Hunan China; 4 Department of Orthopedics, Xiangya Hospital, Central South University, Changsha, 410008 Hunan China; 5 National Clinical Research Center for Geriatric Disorders, Xiangya Hospital, Central South University, Changsha, 410008 Hunan China

**Keywords:** Platelets, Extracellular vesicles, Tumour microenvironment, Engineering, Immunotherapy, Liquid biopsy

## Abstract

Platelets are a significant component of the cell population in the tumour microenvironment (TME). Platelets influence other immune cells and perform cross-talk with tumour cells, playing an important role in tumour development. Extracellular vesicles (EVs) are small membrane vesicles released from the cells into the TME. They can transfer biological information, including proteins, nucleic acids, and metabolites, from secretory cells to target receptor cells. This process affects the progression of various human diseases, particularly cancer. In recent years, several studies have demonstrated that platelet-derived extracellular vesicles (PEVs) can help regulate the malignant biological behaviours of tumours, including malignant proliferation, resistance to cell death, invasion and metastasis, metabolic reprogramming, immunity, and angiogenesis. Consequently, PEVs have been identified as key regulators of tumour progression. Therefore, targeting PEVs is a potential strategy for tumour treatment. Furthermore, the extensive use of nanomaterials in medical research has indicated that engineered PEVs are ideal delivery systems for therapeutic drugs. Recent studies have demonstrated that PEV engineering technologies play a pivotal role in the treatment of tumours by combining photothermal therapy, immunotherapy, and chemotherapy. In addition, aberrant changes in PEVs are closely associated with the clinicopathological features of patients with tumours, which may serve as liquid biopsy markers for early diagnosis, monitoring disease progression, and the prognostic assessment of patients with tumours. A comprehensive investigation into the role and potential mechanisms of PEVs in tumourigenesis may provide novel diagnostic biomarkers and potential therapeutic strategies for treating human tumours.

## Introduction

Platelets are small, non-nucleated cells produced by megakaryocyte shedding in the bone marrow. Platelets have been extensively studied for more than a century, beginning with their discovery and nomenclature, followed by their functional characterisation. Recent research on platelets has focused on platelet-derived extracellular vesicles (PEVs) [1–4]. With scientific and technological advancements and improved research methods, platelet function has been progressively elucidated, from their role in blood coagulation and haemostasis to their ability to regulate inflammatory responses, immune responses, and other pathophysiological processes. Continued abnormal alterations of platelets is closely associated with the development of numerous diseases, including haemophilia, coronary heart disease, aplastic anaemia, and cancer [5–8]. The internal environment in which tumour cells arise and reside is known as the tumour microenvironment (TME). The TME includes immune cells, cancer-associated fibroblasts, endothelial cells (ECs), pericytes, and other cell types that vary between tissues and the extracellular matrix (ECM) [9]. Platelets are a significant component of the immune cell population in the TME [9, 10]. By secreting numerous active biomolecules, they influence other immune cells [11], and they perform cross-talk with tumour cells, thus, playing an important role in tumour development.

Extracellular vesicles (EVs) are membranous nanovesicles that are released from cells and participate in a form of intercellular communication. They can transport ‘cargo’, including DNA, RNA, and proteins, between cells [12, 13]. Although the presence of pericellular vesicles in mammalian tissues or body fluids was first described in the late 1960s, the generic term ‘extracellular vesicles’ was proposed in 2011 to define all extracellular structures encapsulated in lipid bilayers [14]. Initially, EVs were regarded as membrane fragments with no discernible biological significance. However, in 1996, Raposo et al. [15, 16] discovered that EVs could stimulate adaptive immune responses. Since then, the significance of EVs in intercellular communication has been demonstrated in several studies. It is becoming increasingly evident that EVs play a pivotal role in regulating normal physiological processes and form the pathological basis of various diseases. Recent studies have shown that EVs in the circulating human plasma are mainly derived from platelets [17]. PEVs exert a profound influence on tumour progression by regulating the biological behaviour of tumour cells, including malignant proliferation, invasion, metastasis, energy metabolism, and drug resistance. This process is mediated by regulating molecules on the surface of and within tumour cells, including DNA, RNA, and proteins. Consequently, targeting PEVs represents a potentially significant strategy for tumour treatment. Moreover, PEVs exhibit remarkable stability in the bloodstream. The expression levels of PEVs-associated molecules are closely related to the clinicopathological characteristics of patients with tumours. Therefore, PEVs can serve as liquid biopsy markers for tumours and play a significant role in their early diagnosis and monitoring.

We conducted a systematic literature review and analysis of the role of PEVs in tumourigenesis. Here, we summarise the mechanism of action of PEVs in regulating tumourigenesis and tumour progression, we summarise the application of PEVs and their derivatives in anti-tumour therapy, we present an analysis of the association between the expression levels of PEVs-associated molecules and clinicopathological characteristics of patients with tumours, and we discuss the potential and challenges of PEVs as diagnostic biomarkers and therapeutic targets for tumours.

## Platelets

### Discovery and identification of platelets

Human blood contains three main cell types: red blood cells (RBCs), platelets, and white blood cells (WBCs). Under normal conditions, the most abundant blood cells are RBCs, followed by platelets and then WBCs. Platelets are derived from the haematopoietic system and are small, non-nucleated, functional, complex cells formed from megakaryocytes of the bone marrow lineage. They are mainly located within the circulatory system [18]. The chronology of discovering platelets, naming them, and identifying their function spanned several years.

**Fig. 1:**
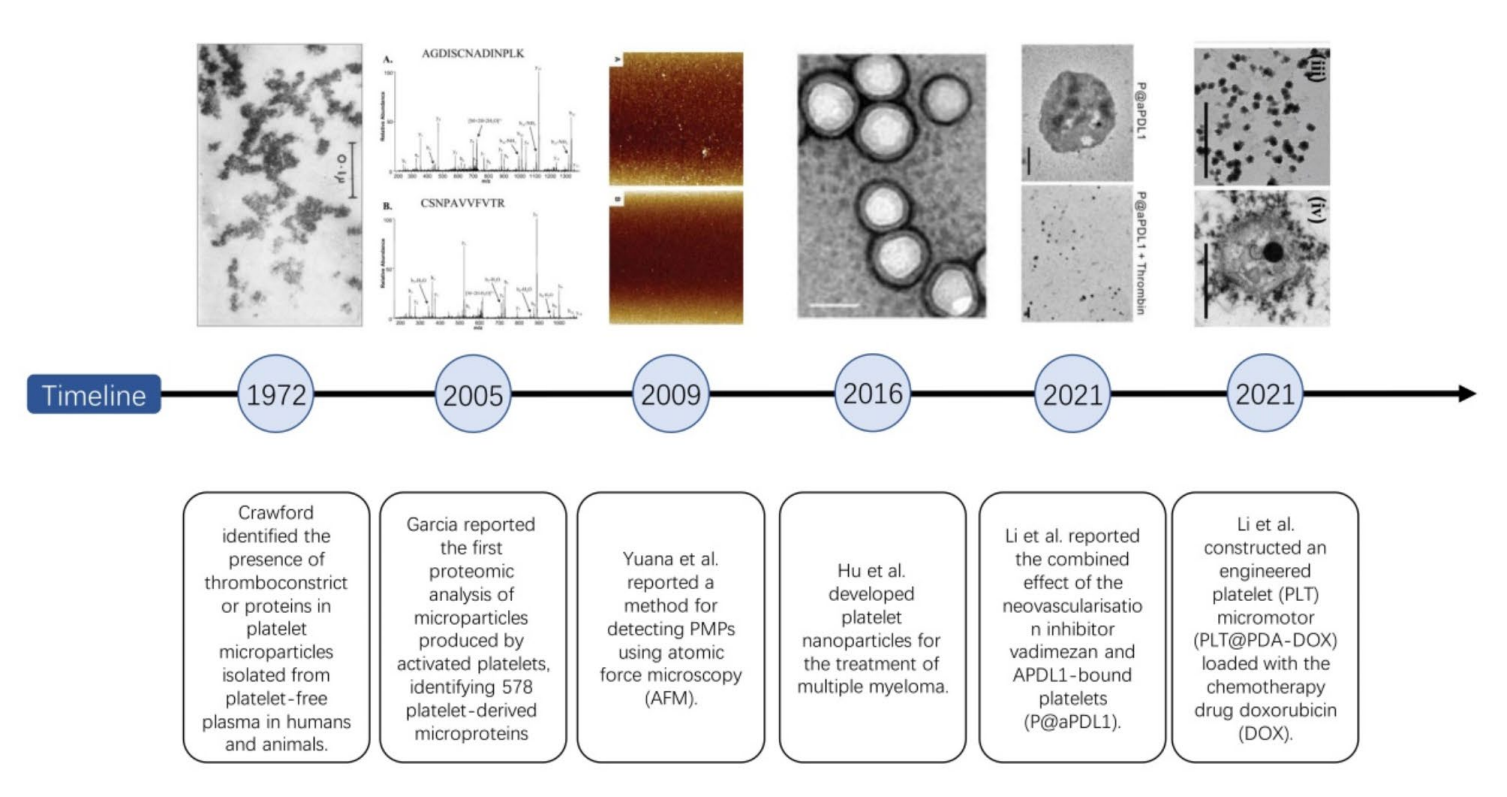
Timeline of milestone events in the research field of platelet-derived extracellular vesicles (PEVs)

Gulliver first observed platelets under a microscope in 1841 and Donne first described platelets as a third particle in the blood in 1842 [4]. In 1882, Bizzozero observed that the initial structure of thrombus formation on the inner surface of an injured blood vessel comprised adhered and aggregated platelets, and he was the first to propose the name ‘platelets’ [19]. In 1906, Wright postulated that platelets are small pieces of cytoplasm shed from megakaryocytes in the bone marrow [20]. Despite the absence of a nucleus, platelets can transport various biomolecules, including proteins and nucleic acids, such as DNA, mRNA, precursor mRNA, and long non-coding RNA (ncRNA) [21]. In 1972, Crawford identified contractile proteins in platelet microparticles isolated from platelet-free human and animal plasma [22]. In 2005, Garcia et al. [23] reported the first proteomic analysis of microparticles produced by activated platelets and identified 578 platelet-derived microproteins using one-dimensional sodium dodecyl sulphate-polyacrylamide gel electrophoresis and liquid chromatography coupled with a linear ion trap mass spectrometer. In 2009, Yuana et al. [24] reported a method for detecting platelet-derived microparticles (PMPs) using atomic force microscopy. In 2016, Hu et al. [25] developed platelet nanoparticles for the treatment of multiple myeloma. In 2021, Li et al. [26] demonstrated the combined effect of the neovascularisation inhibitor Vadimezan and APDL1-bound platelets (P@aPDL1). Subsequently, Li et al. [27] constructed an engineered platelet micromotor (PLT@PDA-DOX) loaded with the chemotherapeutic drug doxorubicin (DOX) (Fig. 1).

### Platelets and human diseases

Platelets contain various proteins, including growth factors, angiogenic factors, chemokines, and immune mediators, etc. [28]. Under normal physiological conditions, platelets help ECs maintain primary haemostasis and intravascular blood flow and play roles in coagulation and tissue repair [29]. Disturbances in platelet levels and function are also closely linked to the onset and development of numerous diseases [30–67] (Table 1), including those characterised by a marked decrease in platelet count, such as aplastic anaemia [30] and acute leukaemia [31–35, 68]; those characterised by platelet dysfunction, such as haemophilia [36–38] and thrombasthenia [69]; and those characterised by an increased platelet count, such as coronary heart disease [52–55] and myocardial infarction [56–67, 70].

**Fig. 2:**
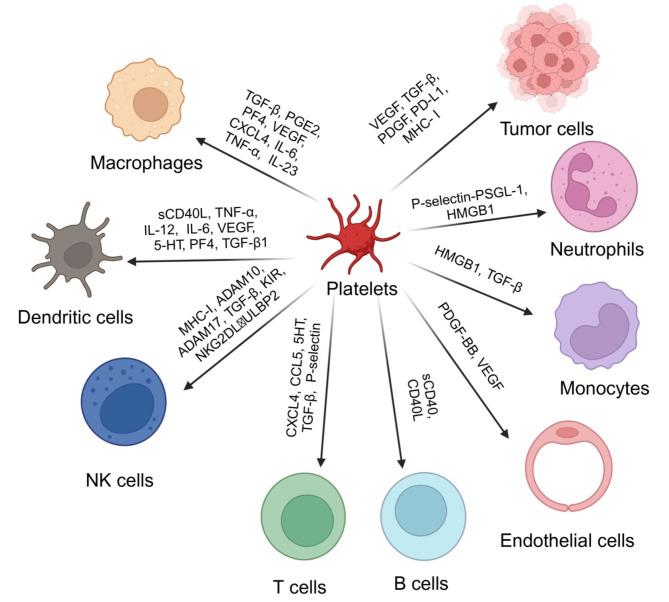
The relationship between platelet-derived extracellular vesicles (PEVs) and cells in tumour microenvironment (TME). TME is a complex and comprehensive system, including tumour cells, stromal cells (e.g. tumor-associated endothelial cells) and immune cells (e.g. macrophages, NK cells, T cells, B cells, DC cells and monocytes). PEVs secrete a variety of cytokines that affect those cells in the TME to influence tumour progression

Platelets also play an important role in cancer regulation. They are important components of the TME [9] (Fig. 2). In addition to affecting other immune cells [11], platelets can act directly on tumour cells. Platelets play important roles in the initiation and development of tumours by communicating with other cells in the TME. For example, they can stimulate tumour angiogenesis and vascular remodelling, protect circulating tumour cells (CTCs) from shear stress and immune surveillance, and promote tumour invasion and metastasis [71]. Platelets can also interact with immune cells in the TME to promote inflammation, tumour angiogenesis, and metastasis [11]. However, tumours can also domesticate platelets, enabling them to capture tumour-derived biomolecules, including mRNA and proteins, and convert platelets into tumour-educated platelets (TEPs) [72].

TEPs contain several active biomolecules, including platelet-specific and circulating biomolecules. These biological substances are released during platelet activation and are involved in the progression of malignant tumours, such as lysosomes, α-granules and dense granules [72]. Tumours can domesticate platelets via both direct and indirect mechanisms. The direct method involves the interaction between platelets and CTCs through several important molecules on the platelet plasma membrane, such as P-selectin, integrins, and glycoproteins. CTCs activate platelets, which in turn contribute to the survival and spread of CTCs. P-selectin is a cell adhesion molecule that is constitutively expressed on the surface of platelets and plays a critical role in the platelet-endothelial response. P-selectin binds directly to CTCs and promotes tumourigenesis. It is also been shown to be upregulated in patients with ovarian cancer. Another mechanism by which cancer cells domesticate platelets involves tumour microvesicles (MVs) and exosomes containing lipids, nucleic acids, and proteins. These vesicles can inhibit the secretion of other molecules and even circulating platelets by binding to P-selectin, thereby domesticating platelets to promote cancer proliferation [73]. TEPs can carry mRNAs and micro RNAs (miRNAs) that help tumour cells enter blood vessels and that bind to tumour cells in the circulation, thus playing an important role in metastasis. In addition, TEPs can help tumour cells resist and evade apoptosis and secrete pro-angiogenic factors, thereby promoting angiogenesis and proliferation [74, 75]. They can also help tumour cells mobilise into blood vessels, produce metastatic foci to enhance the TME and tumour growth, and promote angiogenesis to provide nutrients to the tumour [76]. In addition, TEPs can promote clot formation (thrombosis), similar to platelets, but TEPs may form and consolidate thrombi more readily than platelets [77]. Moreover, TEPs can mediate the communication between distant tumour niches to coordinate indolent tumour outbreaks as overt diseases. TEPs can also mediate the cross-talk between tumours and bone marrow niches to increase bone formation and renewal, a phenomenon associated with the establishment of a pre-metastatic environment [78]. In addition, studies have shown that TEPs can potentially be used as noninvasive biomarkers and can play an important role in early diagnosis, medication decisions, dynamic monitoring, and prognostic evaluation of patients with tumours [71].

**Table 1:** Correlation between abnormal change of platelet and human diseases

Disease Type	Disease conditions	Abnormal change of platelet	Involved molecules or signaling pathways	Therapeutic drugs	Ref.
Aplastic anemia	Dyspnea on exertion, tiredness, easy bruising, ecchymosis, epistaxis, gingival bleeding, menorrhagia, headache, and fever	Decrease (Impaired platelet production)	Thrombopoietin receptor(c-mpl)	Eltrombopag	[30]
Acute leukemia	1) Acute myeloid leukemia: bruising or bleeding, infection, gingival enlargement, leukemia cutis; 2) Acute lymphoblastic leukemia: Discomfort, lethargy, weight loss, fever, night sweats, infection and bleeding, lymphadenopathy, splenomegaly, and hepatomegaly	Decrease (Impaired platelet production)	FLT-3	Midostaurin	[31]
			Hedgehog pathway	Glasdegib	[32]
			BCL-2	Venetoclax	[33]
			IDH2	Enasidenib	[34]
			IDH1	Ivosidenib	[35]
Hemophilia	Bleeding, especially in large joints such as elbows, knees, and ankles	Platelet dysfunction	Absence or complete absence of factor VIII (FVIII) and factor IX (FIX)	Mimics the cofactor function of activated factor (F) VIII	[36]
			Antithrombin	Fitusiran	[37]
			TFPI	Fitusiran	[38]
Lymphoma	Persistent painless lymphadenopathy, Unexplained fever, night sweats, itchy skin, and fatigue	Decrease	BCL6	Peptide inhibitors: BTB binding domain motif peptide Copanlisib Perifosine Everolimus Tazemetostat Ibrutinib Dasatinib Fostamatinib SB1518 Enzastaurin Selinexor	[39]
			PI3K	Small Molecule Inhibitors: 79 − 6	[40]
			AKT	Natural Compounds: Resveratrol	[41]
			mTOR	Copanlisib	[42]
			EZH2	Perifosine	[43]
			BTK	Everolimus	[44]
			LYN	Tazemetostat	[45]
			SYK	Ibrutinib	[46]
			JAK2/STAT	Dasatinib	[47]
			PKC	Fostamatinib	[48]
			XPO1	SB1518	[49]
			PI3K	Enzastaurin	[50]
			AKT	Selinexor	[51]
Coronary heart disease	Typical chest pain symptoms tend to be episodic colic or squeezing pain that begins at the retrosternal or precordial area and radiates upward to the left shoulder, arm, and even the little and ring fingers; some patients have atypical symptoms	Increase (Increased blood viscosity, leading to blockage of blood vessels)	HMGCR	Statins	[52]
			CETP	Anacetrapib	[53]
			PCSK9	Alirocumab	[54, 55]
Myocardial infarction	Chest discomfort with or without dyspnea, nausea, and sweating	Increase (Increased blood viscosity, leading to blockage of blood vessels)	PTEN	HOpic	[56]
			mTOR	Rapamycin	[57]
			NLRP3/caspase-1 inflammasome pathway	16673-34-0	[58]
			TLR4/MyD88/NF-κB-signaling pathway	ApTOLL	[59]
			RhoA/ROCK signaling pathway	Fluvastatin	[60]
			TGF-β/SMADs signaling pathway	Simvastatin	[61]
			Sonic Hedgehog signaling pathway	Erythropoietin	[62]
			Notch signaling pathway	TNF-α inhibitor	[63]
			MAPK signaling pathway	Atorvastatin	[64]
			JAK/STAT signaling pathway	IL-33	[65]
			TGFβ1/TAK1 signaling pathway	Fasudil	[66]
			Hippo/YAP signaling pathway	Luteolin	[67]

## Extracellular vesicles (EVs)

### Biological characterisation of EVs

EVs are secreted by cells and have double-layered phospholipid membrane structures. They carry various biologically active components, including proteins, lipids, and nucleic acids, and mediate intercellular communication in vivo [17, 79, 80]. Based on the biogenesis pathway, EVs can be classified into exosomes, ectosomes, or apoptotic bodies [81, 82]. Exosomes are EVs derived from endosomal systems, and their generation involves double invagination of the plasma membrane and the formation of intracellular multivesicular bodies (MVBs) containing intraluminal vesicles (ILVs). ILVs are finally secreted as exosomes through the fusion of MVBs with the plasma membrane and exocytosis [83]. Exosomes represent a subtype of small EVs, with ILVs of endosomes generally measuring less than 200 nm in diameter. Ectosomes, also known as microvesicles or microparticles, are EVs that germinate directly from the plasma membrane. Various stimuli can shed them, including an increase in intracellular calcium ions [84]. Ectosomes are generally larger than exosomes, with a wide range of sizes [85]. Apoptotic bodies, also called apoptotic vesicles, have a diameter of approximately 100–5,000 nm. They are formed by separating the cytoplasmic membrane from the cytoskeleton due to increased hydrostatic pressure following cell shrinkage [86]. It is worth noting that ‘sEVs’ (representing small EVs) and ‘exosomes’ are not synonymous terms, sEVs include small ectosomes and exosomes.

### Functions of EVs

**Fig. 3:**
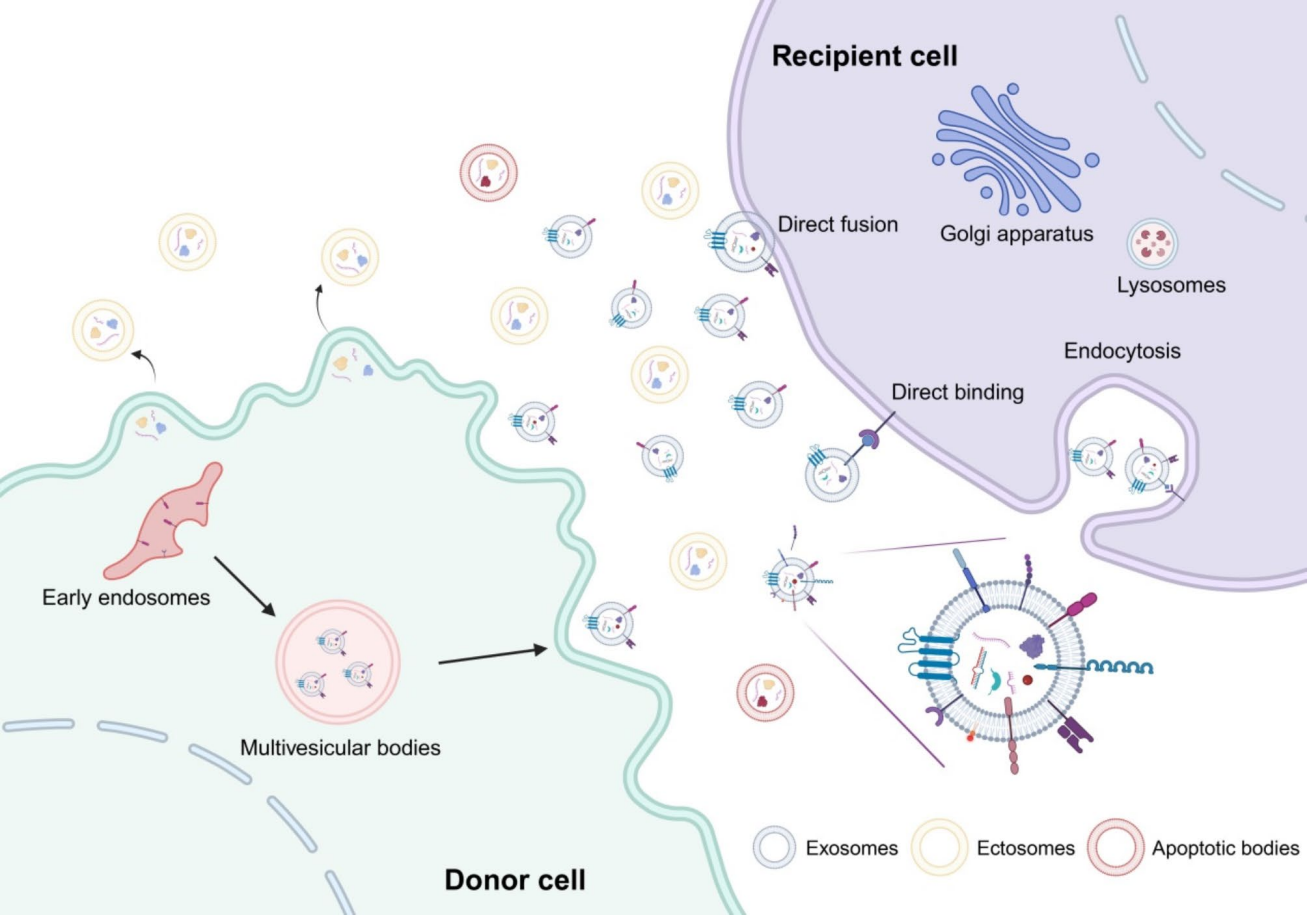
Extracellular vesicles (EVs) biogenesis and secretion in donor cells and communication with recipient cells. EVs participate in intercellular communication by transferring different molecules, including DNA, RNA, lipids, and proteins, etc. EVs act on neighboring or distant receptor target cells through multiple pathways, including direct fusion, endocytosis, and direct binding

Almost all cells in an organism secrete EVs, and numerous studies have shown that EVs are found in various human bodily fluids, including saliva, breast milk, cerebrospinal fluid, ascites, urine, and semen. EVs circulate in the body fluids and act on receptor cells through direct fusion, endocytosis, and binding [14, 87] (Fig. 3). They serve as important carriers of signal transmission, because they carry numerous components, such as proteins, nucleic acids, and lipids, forming a novel intercellular information transmission system that is involved in maintaining various physiological processes in the human body, including stem cell maintenance, tissue repair, immune surveillance, and blood coagulation.

Thus, EVs can be regarded as multifunctional signalling complexes that control basic cellular biological functions. Recent studies have shown that EVs regulate the development of various diseases, particularly tumours. Many studies have demonstrated that, in driving the formation of pre-metastatic tumour ecological niches, EVs can promote tumour progression by inducing cell proliferation, directly stimulating tumour growth, stimulating angiogenesis, secreting matrix proteases to promote matrix remodelling, inducing metastasis, and modulating T cell activity to promote immune escape [15].

### Methods for the isolation and characterisation of EVs

As EVs play important roles in diseases, many researchers have used them in related studies by isolating EVs from immune cells. Based on the physical and biochemical properties of EVs, various separation techniques have been used, such as ultracentrifugation, density-gradient centrifugation, size-exclusion chromatography, ultrafiltration, immunocapture assays, precipitation, and microfluidics. However, none of the above separation methods obtain completely pure samples containing only EVs [88]. Researchers have attempted to use immunomodified superparamagnetic nanoparticles to provide new avenues for rapid, efficient, and high-purity EV separation and elution [89]. The EV identification methods include transmission electron microscopy (TEM), flow cytometry, nanoparticle tracking analysis, and resistive pulse sensing, with identification achieved based on their characteristics, such as morphology, particle size, and surface markers [88, 90–102] (Table 2). Among these methods, flow cytometry, a rapid assay, has the advantages of high-throughput quantitation and multiparameter characterisation. TEM provides high-resolution images of EVs that can distinguish EVs from similarly sized non-EV particles. Atomic force microscopy; microfluidic micronuclear magnetic resonance [102]; and novel optical methods, such as frequency-locked optical whispering evanescent resonators, are also widely applied. Detecting the EV content is an important part of EV functional analysis, and is performed using next-generation sequencing, quantitative PCR, or northern blotting for RNA detection or western blotting, enzyme-linked immunosorbent assays (ELISAs), or mass spectrometry for protein analysis.

**Table 2:** The most common techniques for extracellular vesicles (EVs) isolation

EVs isolation techniques	Methods	Detection range	Advantages	Limitations	Ref.
Transmission Electron Microscopy	The accelerated and aggregated electron beam is projected onto a very thin sample, when the electrons collide with the atoms in the sample, the direction changes, resulting in stereo angular scattering, which can form light and dark images, the image in the magnification, focusing and then displayed on the imaging device.	Capable of detecting EVs as small as 1 nm	Ability to observe EV smorphological features	Inability to represent the particle size distribution and overall concentration of all EVs in the sample	[90, 91]
Flow Cytometry	The particles pass through the laser beam one by one, thus scattering the light and emitting fluorescent signals to multiple measurement channels. The detection of particles is initiated by a signal that exceeds a threshold set on at least one measurement channel.	(1) EVs of 250–500 nm in size; (2) Distinguishes vesicles with a size difference of 200 nm; (3) New generation: 100 nm in size	(1) Fastest detection speed; (2) High-throughput quantitation; (3) Multiparameter characterization	(1) The inability to differentiate accurately between particles and noise; (2) Uncertainty in quantitative detection of EVs	[88, 90, 92–94]
Dynamic Light Scattering	Determines particle size and dispersion by measuring the intensity and intensity distribution of light scattering	EVs of 1 nm to 6 μm in size	(1) Precise, reliable, and repeatable; (2) Sample preparation simple or not required; (3) Easy setup	1) Lower accuracy for suspensions with particles of different sizes (polydisperse suspensions) 2) Inability to provide any biochemical data or information on the cellular origin of the EVs	[94]
Nanoparticle Tracking Analysis	Determines the size and concentration of submicron particles in suspensions by following the Brownian motion of suspended particles with a dark field microscope	EVs of 30 nm to 2 μm in size	(1) Small sample size; (2) High-resolution particle size distribution data and concentration information provided; (3) Enables in-situ testing that is closer to the original state	(1) Inability to differentiate between EVs and non-EV particles; (2) Inability to determine the source of EVs	[88, 90, 95, 96]
Resistive Pulse Sensing	Utilizes the Coulter principle to determine the size and concentration of submicron particles in suspensions	EVs of 70 nm to 10 μm in size	Rapid detection of EV concentration and particle size distribution	(1) Inability to differentiate between EVs and non-EV particles; (2) Difficulty in choosing the right nanopore setup; (3) Pore clogging and pore stability issues; (4) Difficulty in measuring biochemical properties of EVs	[88, 90, 97]
Atomic force microscopy	Works according to the principles of optical and electron diffraction and detects the interaction between the probe tip and the surface of the target sample	Capable of detecting EVs as small as 10 nm to 20 nm	Information on individual EV surface topology, elastic properties, and interaction forces at the supramolecular and submolecular levels provided	(1) EVs need to be stably attached to the substrate; (2) Analysis of EV physical properties requires specialized knowledge	[88, 98]
Mass Spectrographic Analysis	Lonizes the measured substance, separates the ions according to their mass-to-charge ratio, and measures the intensities of various ion peaks for analytical purposes		(1) High-throughput quantification provided; (2) Comparative proteomic analysis of EVs	Long preparation and processing times	[99]
Surface Plasmon Resonance	An optical technology that allows real-time tracking and detection of biomolecules without the need for any markers by immobilizing specific receptors on an active surface coated with gold or silver nanoparticles		(1) High sensitivity; (2) Ability to quantify tumor-derived Evs; (3) No damage to biomolecules		[100]
Electrochemical Sensing	Qualitative or quantitative analysis by determining the electrical or electrochemical properties of the target analyte		1) High sensitivity;2) Quick response; 3) High portability; 4) Economical		[100, 101]
Single Particle Interferometric reflectance Imaging Sensing Analysis	A new technology that combines immunology and optics to capture and separate specific vesicles using immunorecognition, and then quantify the surface markers and contents of the target	Capable of detecting EVs as small as 40 nm	(1) High sensitivity; (2) High precision; (3) Enables fluorescence imaging of individual vesicles		[100]
Microfluidic Micro-Nuclear Magnetic Resonance System Technology	Magnetic nanoparticles are placed in an nuclear magnetic resonance field, where they produce a localized magnetic field that alters the transverse relaxation rate of the surrounding water molecules, resulting in an amplified signal		(1) Simple sample preparation process; (2) High sensitivity; (3) Ability to differentiate cancer-derived EVs from other EVs		[102]

## The underlying molecular mechanism of platelet-derived extracellular vesicles (PEVs) in tumourigenesis

PEVs are the most common type of EVs in the blood [103]. PEVs are subcellular bilayer membrane structures with diameters ranging from 40 nm to 1 μm. They’re produced by platelets after activation or apoptosis. Depending on the biogenesis pathway, PEVs can be classified as platelet-derived exosomes or PMPs, which are also referred to as platelet-derived microvesicles (PMVs) [17]. PMPs are formed by platelet surface shedding, and platelet-derived exosomes originate from the exocytosis of MVBs and α-granules. The exosome-specific marker, tetraspan protein CD63, is particularly abundant in isolated platelet-derived exosomes, whereas annexin-V, factor X, and prothrombin are specific to PMPs [104]. PMPs may play a more important role in procoagulation than platelet-derived exosomes. Other potential markers have been identified on PMPs, including CD31, CD41, CD42a, GPIIb/IIIa, GPIbα, P-selectin, and PF4 [105, 106]. Of these, CD31 and CD41 are the most commonly used markers for identifying PMPs [107]. Generally, PEVs contain various biomolecules, including DNA, RNA, proteins, and small-molecule metabolites. Several databases can be used to analyse the relevant molecules in EVs [108–126] (Table 3). Recent studies have demonstrated that molecules associated with PEVs, such as RNA, lipids, proteins, and DNA, play important roles in the pathogenesis of numerous diseases, particularly cancer.

**Table 3:** Extracellular vesicles (EVs)-associated databases or tools

Database	Species	Extracellular vesicles contents	Website	Ref.
EVmiRNA	Human	microRNA (miRNA)	http://bioinfo.life.hust.edu.cn/EVmiRNA	[108]
exoRBase	Human	Messenger RNA (mRNA), long non-coding RNA (lncRNA), and circular RNA (circRNA)	http://www.exorbase.org/	[109]
ExoCarta	Most species	Protein, mRNA, miRNA, rRNA, tRNA, snoRNA, snRNA, lncRNA, lincRNA, ncRNA, Lipid	http://www.exocarta.org/	[110]
EVpedia	Prokaryotes and eukaryotes	Proteins, mRNAs, miRNAs, lipids and metabolites	https://evpedia.info/evpedia2_xe/	[111]
EMBL-EBI	Human	Proteins	https://www.ebi.ac.uk/QuickGO/	[112]
Vesiclepedia	Most species	Lipids, metabolites, nucleic acids and proteins	http://www.microvesicles.org/	[113]
Urinary Exosome Protein Database	Human	Urinary-associated exosomal proteins	https://esbl.nhlbi.nih.gov/UrinaryExosomes/	[114]
miRandola	Human	Extracellular circulating ncRNA (miRNAs, lncRNAs, circRNAs)	http://mirandola.iit.cnr.it/	[115]
exRNA Atlas	Human and mouse	Small RNA sequencing and qRT-PCR experiment identified extracellular RNA (exRNA) profiles	https://exrna-atlas.org/exat	[116]
EV-TRACK	Most species	EVs-enriched proteins and non EVs-enriched protein	http://www.evtrack.org	[117]
EVAtlas	Human	EVs-assocaited ncRNA(miRNA, snoRNA, piRNA, snRNA, rRNA, tRNA and Y RNA)	http://bioinfo.life.hust.edu.cn/EVAtlas	[118]
ExoBCD	Human	Breast cancer-associated exosomal lncRNA, mRNA and miRNA	http://exobcd.liumwei.org/	[119]
EV-ADD	Human	EVs-associated DNA	https://www.evdnadatabase.com/	[120]
miREV	Human	miRNAs	https://www.physio.wzw.tum.de/mirev/	[121]
ExoceRNA atlas	Human	Competing endogenous RNAs (ceRNAs) in blood exosomes	https://www.exocerna-atlas.com/exoceRNA#/	[122]
CMEP	Human	Circulating miRNA	http://syslab5.nchu.edu.tw/CMEP	[123]
IntiCom-DB	Human	Exosomes-related Inter-tissue communication (ITC) molecules (e.g. Proteins, miRNAs, metabolite)	http://rnanut.net/inticomdb	[124]
SEPDB	Human	Proteins	https://sysomics.com/SEPDB/	[125]
ExplORRNet	Human	miRNA	https://mirna.cs.ut.ee	[126]

### PEVs encapsulate proteins

Protein molecules on the surface or within PEVs play significant roles in tumourigenesis. In patients with nasopharyngeal carcinoma (NPC), PEVs can be transferred into NPC cells and upregulate the expression of integrin β3 (ITGB3). ITGB3 promotes the expression of SLC7A11 by enhancing its stability and activating the MAPK/ERK/ATF4/Nrf2 signalling axis, which inhibits the ferroptosis of NPC cells and promotes their metastasis [127]. Additionally, another study demonstrated that PEVs from patients with colorectal cancer induced Twist1 and vimentin expression in all cancer cell lines studied, whereas PEVs from healthy controls did not. Twist1 plays a significant role in promoting tumour metastasis, tumour initiation, and primary tumour growth, and enhanced vimentin expression contributes to cytoskeletal organisation and adhesion stability, thus enabling cancer cells to resist various stresses generated by the TME and promote malignancy [128]. 12 S-Hydroxyeicosatetraenoic acid (12-HETE), produced by platelet-type 12-lipoxygenase (ALOX12), is a key regulator of tumour metastasis. Another study showed that PEVs can transfer ALOX12 in co-cultures of platelets and HT29 cells (a human colorectal adenocarcinoma cell line) and regulate the invasive metastasis of colon cancer cells [129].

### PEVs encapsulate non coding RNA (ncRNA)

ncRNAs are a class of RNA molecules that are transcribed from the genome, but do not encode proteins. They can be broadly classified into two categories based on their size: small non-coding RNAs (sncRNAs), such as miRNAs, transfer RNA-derived small RNAs, and PIWI-interacting RNAs (piRNAs), and long non-coding RNAs (lncRNAs), such as pseudogenes and circular RNAs (circRNAs) [130, 131]. PEVs can carry ncRNAs, mainly miRNAs, to regulate the occurrence and development of tumours. miRNAs in PEVs either promote or play a role in inhibiting cancer. Liang et al. [132] investigated the role of platelet-derived *miR-223* in regulating lung cancer cell invasion. Compared with healthy subjects, patients with non-small cell lung cancer (NSCLC) show an increased concentration of *miR-223* in platelet-secreted microvesicles. The incubation of human lung cancer A549 cells with platelet-secreted microvesicles results in the rapid delivery of *miR-223* to A549 cells, where platelet-derived *miR-223* targets EPB41L3, thereby promoting A549 cell invasion. EPB41L3 is a member of the band 4.1 family of cytoskeletal proteins. EPB41L3 has been demonstrated to regulate cell shape, intercellular and cell-substrate adhesion, and cell movement, and to participate in the organisation of the actin cytoskeleton. In vitro, MV-mediated transport of platelet *miRNA-let-7a* to human umbilical vein endothelial cells (HUVECs) induces angiogenesis and may stimulate the development of solid tumours [133]. In addition, miRNAs from PEVs play roles in tumour suppression. PMPs transfer *miR-24* to tumour cells both in vivo and in vitro. The direct RNA targets of *miR-24* include mitochondrial *mt-Nd2* and *Snora75*, and their targeting leads to mitochondrial dysfunction and growth inhibition in tumour cells [134].

circRNAs are particularly abundant in platelets compared with other haematopoietic cell types, and they can be packaged and released into PEVs by platelets [135]. Preußer et al. [135] found that circRNAs were selectively released into vesicles and they observed a subset of targets (AMD1 and DYRK1A for microvesicles and FAM13B, DYRK1A, AMD1, and TMEM30 for exosomes) that were preferentially released into PEVs compared with their corresponding linear isoforms, and this process may have involved specific sorting mechanisms. Additionally, researchers have identified an abundant platelet-specific circRNA (*Plt-circR4*) that is not preferentially released into vesicles, supporting the selective release of RNAs. circRNAs may also be involved in signalling pathways and play a role in cancer progression.

### PEVs encapsulate other molecules

In addition to proteins and nucleic acids, EVs typically contain lipids, small molecule metabolites, and mitochondria. It has been reported that the PMP subgroup retains functional mitochondria [136]. PEV-derived mitochondria can be transferred to tumour cells to regulate their metabolic function. Using flow cytometry, fluorescence microscopy, and quantitative real-time fluorescence PCR, Gharib et al. [137] confirmed that platelet-derived mitochondria were transferred to chronic lymphocytic leukaemia (CLL) cells and that this affected the metabolic reprogramming of tumour cells and supported phenotypic changes during the malignant process.

## PEVs in cancer progression

**Fig. 4:**
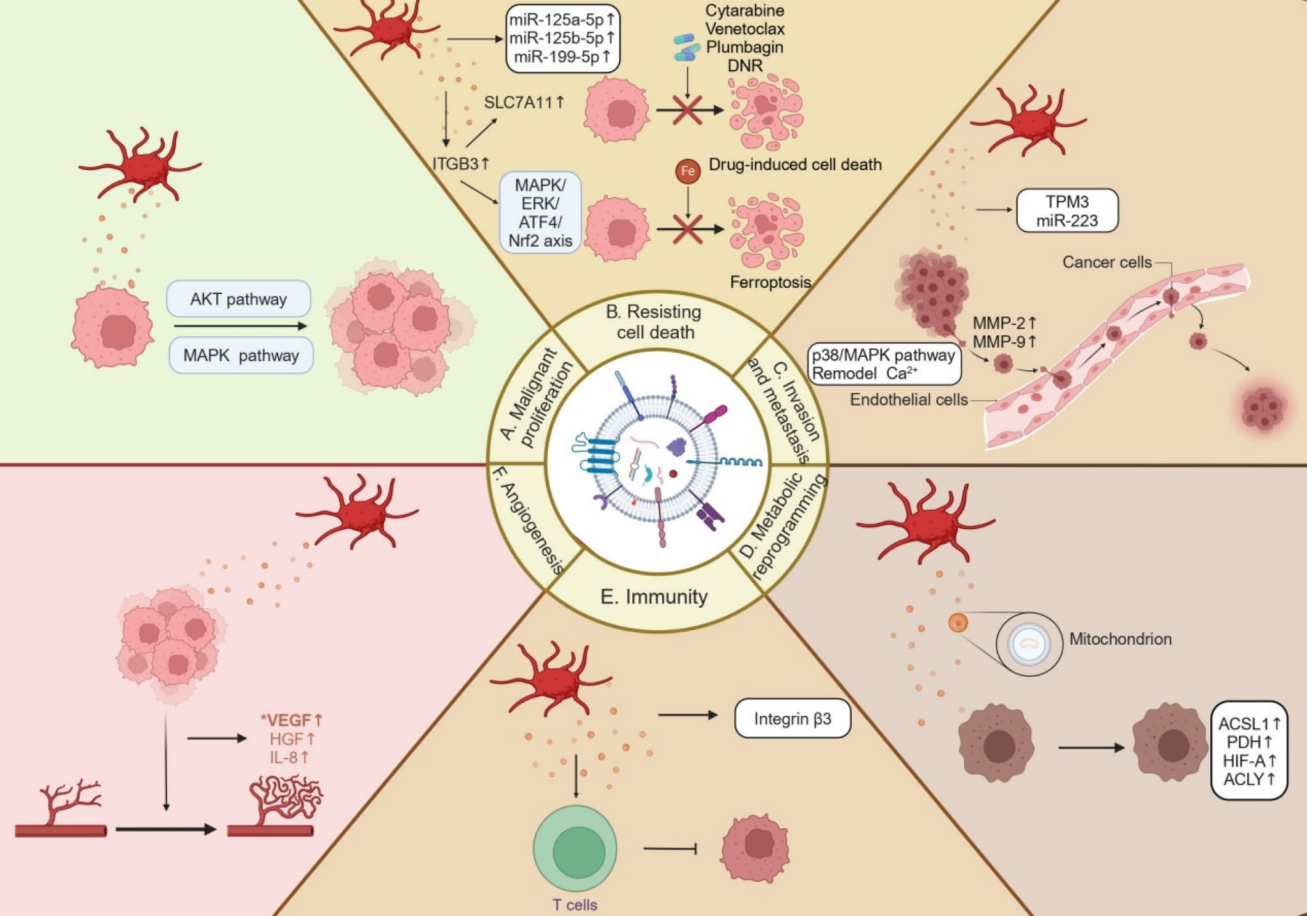
Platelet-derived extracellular vesicles (PEVs) regulate the progression of tumours. (**A**) PEVs promote malignant proliferation of tumour cells by activating the MAPK and AKT pathways of tumour cells. (**B**) PEVs promote tumour cell resistance to drug-induced cell death by regulating the increase of miRNAs (such as miR-125a-5p, miR-125b-5p, and miR-199-5p) in tumour cells. PEVs also regulate ITGB3 protein to promote the expression of the SLC7A11 protein, thereby activating the MAPK/ERK/ATF4/Nrf2 signalling axis which inhibits ferroptosis in tumour cells. (**C**) RNA (such as TPM3 mRNA and miR-223) secreted by PEVs can regulate the invasion and metastasis of tumour cells. It can also affect the increase of protein MMP-2 and MMP-9 in tumour cells through Ca^2+^ remodelling and the p38/MAPK pathway, thus leading to the invasion and metastasis of tumour cells. (**D**) Mitochondria secreted by PEVs regulate tumour cell metabolism through proteins (ACSL1, PDH, HIF-A, and ACLY), further promoting cell glycolysis. (**E**) The secretion of integrin β3 by PEVs affects the immune responses of T cells to tumour cells. (**F**) PEVs affect the angiogenesis of tumour cells by regulating the increase of angiogenesis-related molecules (VEGF, HGF, and IL-8) in tumour cells

Studies have shown that PEVs participate in regulating tumourigenesis and tumour development, affecting various malignant biological phenotypes, including malignant proliferation, resistance to cell death, invasion and metastasis, metabolic reprogramming, immunity, and angiogenesis (Fig. 4).

### Role of PEVs in the malignant proliferation of tumours

The initial impression of a tumour is that it can replicate indefinitely, that is, it undergoes malignant proliferation. Tumour cells receive sustained proliferative signals in various ways. For example, they produce their own growth factor ligands and respond by expressing homologous receptors, resulting in autocrine proliferative stimulation. Tumour cells can maintain telomere DNA for an extended period, thus preventing the onset of senescence or apoptosis. This is typically achieved by upregulating telomerase expression, although it can also be achieved by upregulating telomerase expression to a lesser extent [138].

The proliferation of tumours is inhibited by PEVs, which affect the cell cycle. By incubating and culturing lung cancer A549 cells with PMVs and exosomes, respectively, Wieczorek et al. [139] found that PMVs and exosomes activate the MAPK^p42/44^ and AKT pathways and upregulate cyclin D2 to promote the proliferation of A549 cells in serum-free culture medium. Gharib et al. [137] used co-culture experiments involving PMPs and CLL cells (CII and MEC-1) to demonstrate that PMPs directly affect the proliferation of CLL cells by internalising the mitochondria and enhancing mitochondrial activity. The results showed that the effect of PMPs on the proliferation of cancer cells was significantly influenced by the health status of PMP mitochondria. Additionally, PMPs facilitated the accelerated growth of CLL cells following internalisation. Furthermore, Vismara et al. [140] cocultured PEVs with various types of breast cancer (BC) cell lines and discovered that PEVs could alter the cell cycle progression of BT474 and SKBR3 cells by altering the intracellular calcium concentration.

### Role of PEVs in tumour cell death resistance

To define cell death, the Nomenclature Committee for Cell Death has previously proposed three criteria for identifying dead cells: (1) permanent loss of plasma membrane barrier function; (2) the disintegration of cells into discrete fragments, commonly referred to as apoptotic bodies; or (3) the engulfment of cells by phagocytes or other cells with phagocytic activity [141]. Some cancer cells do not undergo cell death after receiving the appropriate cell death signal, but evade this process by exhibiting resistance to cell death. Tumour cells use various strategies to limit or avoid cell death. The mechanisms of cell death are diverse, including intrinsic apoptosis, extrinsic apoptosis, ferroptosis, pyroptosis, autophagy-dependent cell death, immunogenic cell death, and cellular senescence [142]. Apoptosis is the most common mechanism; however, ferroptosis and immunogenic cell death are the latest mechanisms identified through advances in cell death research and are of great importance in the study of tumour resistance to cell death.

PEVs inhibit the ferroptosis of tumour cells and induce tumour cells to resist drug-induced cell death. When platelets interact with cancer cells, they internalise PEVs to transfer the substances carried by PEVs to the cancer cells and promote resistance to cell death [138]. Various in vivo and in vitro experiments conducted by Li et al. [127] demonstrated that PEVs in patients with NPC could be transferred into NPC cells and upregulate ITGB3 in these cells. ITGB3 increase the protein levels of SLC7A11 by enhancing its stability, thereby activating the MAPK/ERK/ATF4/Nrf2 signalling axis, which inhibits ferroptosis in NPC cells. Moreover, PEVs can promote the resistance of cancer cells to drug-induced cell death. Gharib et al. [137] used an apoptosis assay to demonstrate that the enhancement of PMP-mediated mitochondrial internalisation and oxidative phosphorylation in CLL cells improved the resistance of these cells to the anticancer drugs cytarabine, Venetoclax, and plumbagin. Cacic et al. [143] demonstrated that the expression levels of *miR-125a-5p*, *miR-125b-5p*, and *miR-199-5p* were elevated in THP-1 cells following the co-incubation of PMPs with THP-1 cells. Moreover, PMPs reduced the mitochondrial membrane potential in THP-1 cells, inhibited cell cycle progression, reduced cell proliferation, and induced their differentiation into macrophages, thereby increasing the drug resistance of the cells to daunorubicin. Therefore, platelet inhibitors are a potential therapeutic strategy for acute myeloid leukaemia.

### Role of PEVs in tumour invasion and metastasis

The multistep process of invasion and metastasis is schematised as a series of discrete steps, often referred to as the invasion-metastasis cascade. It involves a series of cellular biological changes, starting with local invasion, followed by the entry of cancer cells into nearby blood and lymphatic vessels; the transport of cancer cells through the lymphatic and haematopoietic systems; the escape of cancer cells from the vascular lumen into the parenchyma of distant tissues (extravasation); the formation of small nodules of cancer cells (micrometastasis); and finally, the growth of micrometastatic foci into tumours that are visible to the naked eye, in a step that has been termed ‘colonisation’ [144, 145].

PEVs are involved in the process of tumour cells acquiring the ability to grow invasively and metastasise by affecting various molecules, such as integrins, metalloproteinases, Ca^2+^ ions, and ncRNAs. Kassassir et al. [146] found that PMPs can be bound to colorectal cancer cells by transferring integrins, by stimulating the expression of integrin subunits already present on the surface of the colorectal cancer cells, and by stimulating the expression and activity of matrix metalloproteinases (MMP)-2 and MMP-9 in colorectal cancer cells through the p38/MAPK pathway, thereby increasing epithelioid and mesenchymal-like colorectal cancer cell mobility and invasiveness. Additionally, PEVs effectively stimulate the migration and invasion of breast cancer (BC) cells. They induce a sustained increase in the intracellular Ca^2+^ concentration in MDA-MB-231 BC cells, which is associated to the stimulation of selected signaling proteins implicated in migration, including and myosin light chain and p38 MAPK [140]. In addition, Vismara et al. [147] showed that PEVs stimulate migration by partially remodelling the Ca^2+^-handling machinery in MDA-MB-231 cells. Moreover, platelet *TPM3* mRNA may be delivered to BC cells via microvesicles, leading to an enhanced migratory phenotype of the cells [148]. Liang et al. [132] showed that, following the co-incubation of human lung cancer A549 cells with PMVs, the PMVs rapidly introduced *miR-223* into the cells, which targeted EPB41L3, thereby promoting the invasion of A549 cells.

### Role of PEVs in the metabolic reprogramming of tumour cells

Metabolic reprogramming refers to the phenomenon in which tumour cells adapt to a hypoxic, and nutrient-deficient microenvironment, and proliferate rapidly by changing their metabolic patterns to regulate their cellular functions and help them resist external stresses to meet their own growth and energy needs [149]. Metabolic reprogramming is an important factor that affects cancer development and progression. The main modalities include glycolysis; oxidative phosphorylation; and amino acid, fatty acid, and nucleotide metabolism.

PEVs can influence metabolic reprogramming in tumours through the mitochondria. Gharib et al. [137] found that, after co-culturing CLL cells with PMPs, the transfer of the mitochondrial contents and function provided by PMPs induced metabolic reprogramming, leading to an increase in the oxygen consumption rate, ATP levels, and reactive oxygen species production. Further exploration revealed that PMPs increased the expression levels of metabolic genes associated with CLL progression. Bioinformatic techniques were used to analyse the mechanisms involved in metabolic reprogramming, resulting in the identification of 17 genes that exhibited significant alterations during disease progression. These included hypoxia-inducible factor-alpha (*HIF-A*), ATP citrate lyase (*ACLY*), long-chain fatty acyl-CoA ligase (*ACSL1*, *3*, and *4*), pyruvate dehydrogenase (*PDH*), and fatty acid synthase (*FASN*). These genes are involved in metabolic reprogramming by regulating metabolic pathways, such as NF-kB and survival signals (R-HSA-209560), which are highly activated in the early stages of CLL and by regulating ChREBP-activated metabolic gene expression (R-HSA-163765), which plays a central role in advanced CLL. Furthermore, Cereceda et al. [150] showed that platelet-derived mitochondria could be internalised by triple-negative breast cancer (TNBC) MDA-MB-231 cells via EVs to enhance their metabolic adaptation.

### Role of PEVs in tumour immunity

As a key component of the TME, immune cells can either promote or inhibit tumorigenesis [151]. Innate immune cells play significant roles in tumour progression and have a potential tumour-promoting effect. Inflammation can promote numerous functions by providing biologically active molecules to the TME, including growth factors, which maintain proliferative signals; survival factors, which limit cell death; proangiogenic factors; extracellular-matrix-modifying enzymes, which promote angiogenesis; and invasive, metastatic, and inducible signals, leading to the activation of epithelial-mesenchymal transition (EMT). Furthermore, most early-stage tumour cells are recognised and cleared by the immune system, and solid tumours somehow avoid detection by various branches of the immune system or limit the extent of immune killing, thus avoiding eradication [138].

PMPs exert pro-inflammatory and immunosuppressive effects that may influence tumour progression. Zhou et al. [152] found that integrin β3 deficiency in hepatocellular-carcinoma-infiltrating T cells may be involved in forming an immunosuppressive TME. Furthermore, cell co-culture and flow cytometry demonstrated that PMPs overtly transferred integrin β3 to T cells and promoted T cell infiltration and Th1-type immune responses, mediated through clathrin-dependent endocytosis and macrophage phagocytosis. One reason why PMV-neutrophil interactions promote neutrophil activation is that PMVs transfer glycoprotein IIb/IIIa receptors to neutrophils and activate them via the NF-κB pathway [153]. It has been demonstrated that some platelet-derived mitochondria contain respiratory-competent mitochondria. These platelet-secreted mitochondria are substrates for type IIA-secreted phospholipase A2, which produces inflammatory mediators, such as lysophospholipids, fatty acids, and mitochondrial DNA (mtDNA), and promotes leukocyte activation [136]. Kuravi et al. [154] demonstrated that PEVs facilitate the adhesion of mobile neutrophils to ECs. Sprague et al. [155] first demonstrated that PEVs could deliver CD154, stimulate antigen-specific IgG production, and regulate germinal centre formation by reacting with CD4^+^ T cells. Ren et al. [156] investigated the potential association between PMPs and inflammatory cytokine secretion in oral squamous cell carcinoma (OSCC). Using ELISAs, researchers found that plasma interleukin-6 (IL-6) and TNF-α levels were significantly higher in patients with OSCC than in healthy subjects. Furthermore, correlation analysis showed that plasma IL-6 and TNF-α levels were positively correlated with the number of PMPs in OSCC. Moreover, immunohistochemistry results were in high concordance with the ELISA results, indicating that the elevated number of PMPs was closely related to the secretion of inflammation-related cytokines. However, further studies are required to elucidate the role of PMPs in the immune response in the TME and their impact on anti-tumour therapy.

### Role of PEVs in tumour angiogenesis

Tumours, like normal tissues, require nutrient and oxygen support and the ability to excrete metabolic waste and carbon dioxide. Tumour-associated neovessels generated by angiogenic processes satisfy these criteria. During tumour progression, an angiogenic switch is almost always activated and remains open, such that a normally quiescent vascular system grows new blood vessels constantly to help sustain tumour growth [138].

PEVs exert a strong pro-angiogenic effect by affecting angiogenic factors. Using real-time reverse transcription-PCR and other techniques, Janowska-Wieczorek et al. [139] found that PMVs stimulated an increase in the mRNA expression levels of angiogenic factors produced by human lung cancer cells, such as vascular endothelial growth factor (*VEGF*), hepatocyte growth factor, and *IL-8*. Anene et al. elucidated the mechanism of PMP-induced angiogenesis [133]. Using co-culture assays, luciferase reporter assays, and antisense oligonucleotide technology, they demonstrated that the PMP-derived miRNA *Let-7a* could be delivered to HUVECs and inhibit expression of the anti-angiogenic factor THBS-1 by directly binding to the 3′ untranslated region of *THBS-1* mRNA, thereby promoting angiogenesis.

## PEVs can be used as novel tumours biomarkers

With the rapid development of high-throughput sequencing technologies, liquid biopsies have become increasingly important in precision oncology. Liquid biopsy is a novel technique that detects tumour-associated molecular markers in specimens by analysing CTCs, circulating tumour DNA, circulating free DNA or RNA, exosomes, TEPs, circulating tumour-derived endothelial cells, and protein molecules [157]. TEPs, as a class of tumour-educated platelets, are important liquid biopsy markers that are highly abundant; easily isolated from blood; and rich in DNA, RNA, and proteins [157]. The protein and RNA profiles of TEPs change dynamically in patients with different types of cancer, even during the early stages of disease. The combination of liquid biopsies with high-throughput sequencing technologies (particularly single-cell sequencing [158]) has been widely used in recent years [157]. Numerous studies have shown that PEVs (especially PMPs) can be used as biomarkers for early diagnosis, monitoring disease progression, and prognostic assessments of various tumours [148, 159–165] (Table 4).

**Table 4:** Correlation between platelet-derived extracellular vesicles (PEVs) and clinicopathologic features and therapeutic outcomes of tumour patients

Tumour types	PEV contents	Treatment	Relationship with therapeutic outcomes	Relationship with clinicopathologic features of tumour patients	AUC	Ref.
Breast cancer	TPM3 mRNAs			Tumour metastasis	0.8404 (95% CI, 0.7566–0.9242, *p* < 0.001)	[148]
				Tumour grading; tumour metastasis; tissue-based biomarkers (ER, PR, Her-2)		[159]
NSCLC		Immunotherapy (pembrolizumab or nivolumab) ± chemotherapy	PMPs ≥ 80 events/μL were associated with disease progression in advanced NSCLC and independently predicted the therapeutic efficacy of immunotherapy		0.836 (95%CI,0.724–0.948, *p* < 0.0001)	[160]
		Chemotherapy; immunotherapy; target therapy	PMPs ≥ 80 events/μL were independently associated with the progression of advanced NSCLC		0.804 (95%Cl,0.706–0.903, *p* < 0.0001)	[161]
		Chemotherapy ± immunotherapy (bevacizumab)		Biomarkers (HMGB1, PAI-1)		[162]
		Target therapy; chemotherapy		Prognosis	PDAc-MPs (time 3): 0.659 (95%Cl, 0.760–0.907, *p* < 0.001); PDAp-MPs (time 3): 0.707 (95%Cl, 0.667–0.847, *p* = 0.001)	[163]
Hormone-refractory prostate cancer		Docetaxel-based chemotherapy		Prognosis		[164]
Pancreatic ductal adenocarcinoma		Surgery	PEVs (CD41^+^-EVs and CD61^+^-EVs) reflected tumour burden before and after surgical resection		CD41^+^-EVs: 0.678 (95%Cl,0.552–0.804); CD61^+^-EVs: 0.652 (95%Cl,0.527–0.776)	[165]

Note: NSCLC, non-small cell lung cancer; AUC, area under the curve; PDAc-MPs, platelet-derived activated microparticles; PDAp-MPs, platelet-derived apoptotic microparticles

### PEVs can be used as novel biomarkers for the early diagnosis of tumours

Accurate and early detection of cancer is critical, and there is an urgent need to develop novel biomarkers to enable early diagnosis. EVs circulating in the peripheral blood are potential non-invasive biomarkers to complement current clinical approaches for the early detection of cancer. As one of the most common types of microparticles, the role of PMPs in cancer diagnosis has been increasingly emphasised. Haghbin et al. found that the number of circulating PMPs was higher in patients with BC than in healthy controls, and the number of PMPs was significantly correlated with tissue biomarkers, such as oestrogen receptor (ER), progesterone receptor (PR), and human epidermal growth factor receptor 2 (Her-2) [159]. In addition, Odaka et al. [165] found that the number of serum PEVs (CD41^+^-EVs and CD61^+^-EVs) was significantly higher in patients with pancreatic ductal adenocarcinoma (PDAC) than in healthy controls and that the areas (AUCs) under the receiver operating characteristic (ROC) curves of CD41^+^-EVs and CD61^+^-EVs were 0.678 and 0.652, respectively, indicating that they performed moderately well as diagnostic markers and could assist in diagnosing PDAC. Recently, artificial intelligence (AI) has gradually been used for the early diagnosis of tumours [166]. The combined application of AI technology with platelets and related molecules in tumour detection and other areas is expanding. Best et al. [167] demonstrated that particle-swarm optimisation–enhanced algorithms were effective at selecting RNA biomarker panels from a platelet RNA sequencing library (*n* = 779), enabling the accurate TEP-based detection of early and advanced NSCLC (late-stage validation cohort [*n* = 518]: accuracy, 88%; AUC, 0.94; 95% confidence interval [CI], 0.92–0.96; *p <* 0.001; early-stage validation cohort [*n* = 106]: accuracy, 81%; AUC, 0.89; 95% CI, 0.83–0.95; *p <* 0.001). Furthermore, TEP RNA biomarkers distinguish patients with NSCLC from healthy individuals and those with non-cancerous inflammatory conditions. Xie et al. [168] developed a machine-learning-based radiomics model to predict extrapancreatic extension (EPE) and selected the most appropriate classifier, extreme gradient boosting (XGBoost), to construct the model. Platelets were identified as the most relevant clinical factor using a function known as SelectKBest, and were added to the combined model, which has the potential to noninvasively and accurately predict EPE preoperatively, aiding in pancreatic cancer treatment. In another study, the prognostic value of platelet-derived growth factor receptor β was validated using an AI model (Aiforia^®^Platform), further confirming it as a potential marker for risk stratification and treatment of malignant pleural mesothelioma [169]. AI combined with PMPs has a promising future in studying assisted tumour diagnosis, but more studies on this topic need to be conducted.

### PEVs can be used as novel biomarkers for monitoring tumour progression

Disease progression refers to the change in a patient’s condition, usually after disease onset. There is a lack of a ‘gold standard’ for monitoring disease progression in treating diseases, especially tumours. Studies have shown that PMPs can be used to monitor cancer progression. Haghbin et al. [159] found that, in patients with BC, the number of PMPs was significantly higher in patients with larger tumour sizes (T2) (750 ± 356/μL; *p* < 0.001) than in patients with smaller tumour sizes (T1) (401.1 ± 198.8/μL) and controls (217.4 ± 108.6/μL), and that the number of PMPs was significantly correlated with tumour grading and tumour metastasis. In addition, Odaka et al. [165] found that number of serum PEVs (CD41^+^ EVs and CD61^+^ EVs) were significantly lower in patients with PDAC after surgical resection; thus, the number of CD41^+^ EVs and CD61^+^ EVs may reflect the tumour burden. Furthermore, we examined whether the preoperative number of EVs could predict PDAC recurrence, and the results indicated that the recurrence group exhibited a trend toward more CD41^+^-EVs and CD61^+^-EVs than the non-recurrence group, although this was not statistically significant. Moreover, the AUC values of CD41^+^-VEs and CD61^+^-EVs in early PDAC (0.685 and 0.657, respectively) were similar to those in late PDAC (0.668 and 0.644, respectively), suggesting that the number of serum PEVs was similar in early and late PDAC.

### PEVs can be used as novel biomarkers for prognostic assessments of tumours

One of the current difficulties in clinical research is identifying markers for clinical use to monitor treatment efficacy, recurrence, and metastasis, and evaluate the prognosis of malignant tumours. Recent studies have shown that PMPs play an important role in the prognostic assessment of various tumours. In a study analysing circulating PMPs after treatment with immune checkpoint inhibitors in patients with advanced NSCLC, the number of PMPs was significantly higher in the immune-related disease progression group than the immune-related objective response group. It was concluded that PMPs independently predicted the therapeutic effect of immunotherapy. The ROC curve showed an AUC of 0.836 (95% CI, 0.724–0.948, *p* < 0.0001), a sensitivity of 88.9%, a specificity of 71.9%, a positive predictive value of 64.0%, and a negative predictive value of 92.0% when PMPs were used to predict the effects [160]. In a similar study by Liu et al. [161], 86 patients with NSCLC were included. Multivariate logistic regression analysis showed that a PMP concentration ≥ 80 events/μL (odds ratio [OR], 10.968; 95% CI, 2.973–40.462, *p* < 0.0001) was independently associated with the progression of advanced NSCLC and that predictive models of disease progression using neutrophil microparticles and PMPs combined with the neutrophil/lymphocyte ratio were effective at identifying 80.8% of patients with disease progression. Niki et al. [162] found that the number of PMPs in NSCLC correlated with the levels of high-mobility group box-1 (HMGB1) and plasminogen activator inhibitor-1 (PAI-1), and that the combined increase in all three parameters was associated with the prognosis of NSCLC. Similarly, Yamanaka et al. [170] found that the combined increase in the number of PMPs and the levels of HMGB1 might be associated with cancer-associated thrombosis, leading to a poor prognosis. A study by Wang et al. [163] confirmed significantly higher concentrations of platelet-derived activated microparticles (PDAc-MPs) and platelet-derived apoptotic microparticles (PDAp-MPs) in PMPs in the third month after treatment in the disease progression group than the disease control group (circulating concentrations of PDAp-MPs and PDAc-MPs were > 15,055 counts/mL and 62,700.5 counts/mL, respectively), suggesting that PDAc-MPs and PDAp-MPs may be useful biomarkers for predicting prognostic outcomes in patients with NSCLC. Helley et al. [164] studied patients with hormone-refractory prostate cancer and found that patients with a PMP concentration > 6,867/μL in whole blood had a significantly shorter median overall survival (OS) time than patients with lower PMP concentrations (16.7 months versus 26.4 months, *p* = 0.034 [Breaslow-Gehan test]), and a statistically significant interaction term between PMPs and platelets was significantly associated with OS (*p* = 0.019). In addition, a study analysing patients with upper gastrointestinal malignancies undergoing abdominal surgery demonstrated increased PMP formation at high preoperative shear stress (*p <* 0.0001), which was further enhanced on postoperative day 1 (*p* = 0.0012), suggesting that surgical intervention may increase the risk of thrombotic complications [171].

## PEVs are potential therapeutic targets of cancer

Since platelet activation is closely related to tumour progression, inhibiting platelet activation is one way to inhibit tumour progression. Traditional therapeutic targets for inhibiting platelet activation mainly include cyclooxygenase-1, integrin αIIbβ3, and the P2Y12 receptor [172]. However, platelet-derived nanostructures or microstructures, such as engineered platelets and platelet membrane (PM)-encapsulated nanoparticles, are emerging as potential drug-delivery systems in cancer therapy, due to the natural binding and accumulation properties of platelets to the tumour site; thus, they can achieve better tumour targeting than conventional chemically synthesized nanocarriers [173].

### PEVs and PEVs-associated nanoparticles as carriers for drug delivery

Targeting drugs to specific sites of action via nanocarriers has opened a new treatment paradigm [174]. PEVs and platelet-derived nanoparticles represent promising avenues for developing novel drug-delivery systems with significant therapeutic potential. PEVs, which are lipid vesicle nanocontainers loaded with bioactive molecules [175], and platelet-derived nanoparticles, which are nanostructures that utilise platelet membranes to cover the synthesised nanoparticles [176], are relatively similar in nature, with both exerting their targeting effects on the PM surface.

PEVs have attracted considerable attention as promising carriers for drug delivery. There are two general drug-loading strategies for PEVs: one based on preloading the drug into platelets, immediately followed by the production of PEVs, and the other based on the post-loading of isolated PEVs. The first strategy was reported by Zhang et al. [177] who showed that cyclophosphamide-loaded PD-1-presenting platelets, by releasing PD-1-modified cyclophosphamide-loaded PMPs, blocked the immunosuppressive effects of PD-L1 on tumour cells, depleted regulatory T cells (Tregs), and promoted the appearance of CD8^+^ lymphocytes in the TME. In the second strategy, Ning et al. [178] designed platelet-exosome-hybridised liposome nanoparticles co-loaded with aggregation-induced emission photosensitizers and chloroperoxidase to promote uninterrupted single-linear oxygen production, thereby enhancing BC immunotherapy.

The application of platelet-derived nanoparticles in anti-tumour therapy has also been increasingly investigated. Shang et al. [179] constructed a nanoplatform of zirconia nanoparticles (ZrO2 NPs) coated with a platelet membrane (PLT@ZrO2) that significantly inhibited tumour growth, invasion, and metastasis. Zhuang et al. [180] reported a platelet-cell-membrane-coated metal-organic framework nanodelivery platform for the in vivo targeted delivery of small interfering RNAs (siRNAs). Significant anti-tumour targeting and therapeutic efficacy were observed in a mouse xenograft model using this platform. In addition, nanoscale vesicle systems based on platelet microparticles have been developed, such as platelet-microparticle-inspired nanovesicle (PMIN) systems, which can be targeted for drug delivery [175] and may be applied to anti-tumour therapy in the future.

### Potential application of PEVs and PEVs-associated nanoparticles in anti-tumour therapy

**Fig. 5:**
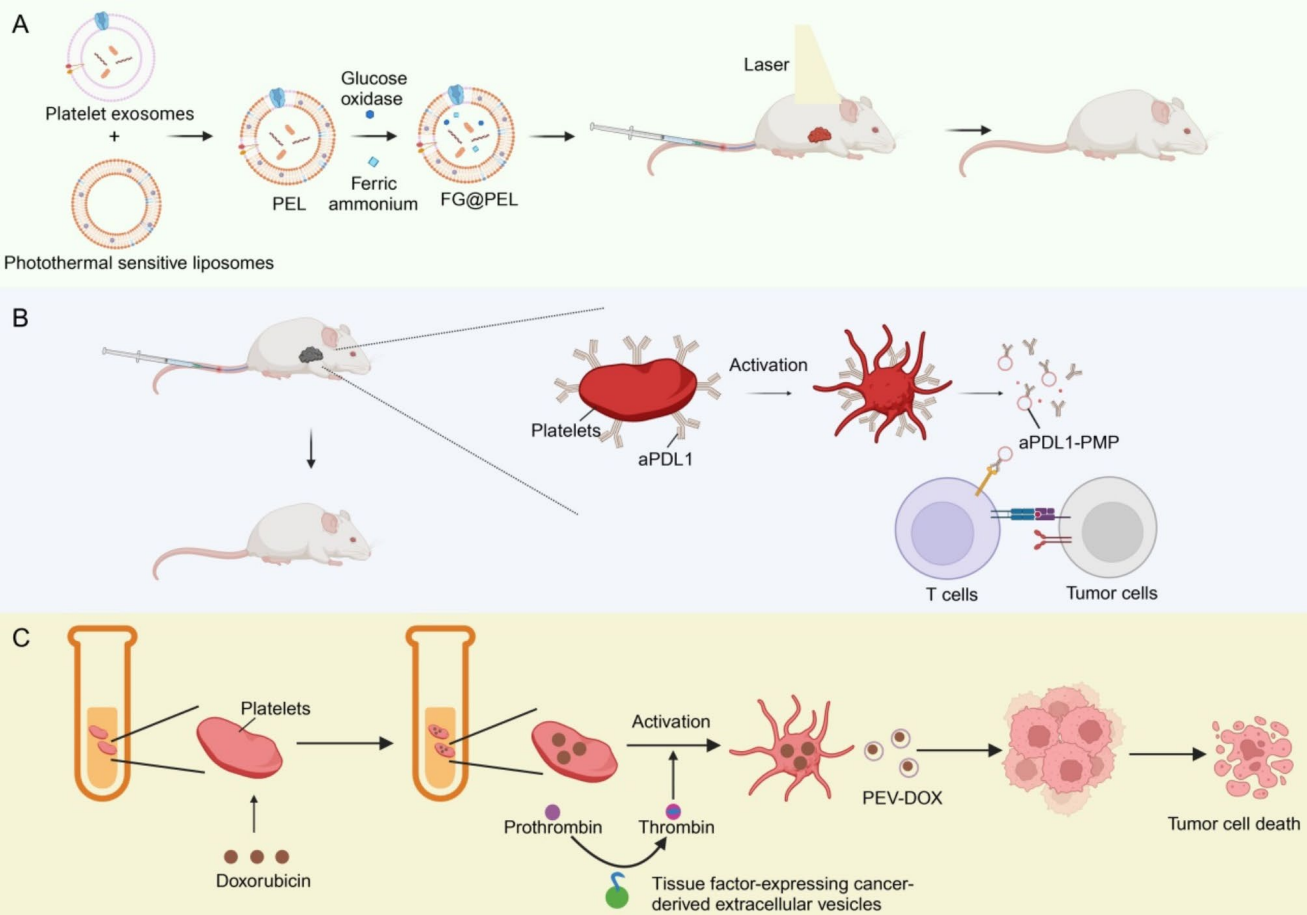
The potential application of platelet-derived extracellular vesicles (PEVs) in the targeted therapy of cancer. (**A**) PEVs combined with photothermal therapy. Combining platelet exosomes with photothermal sensitive liposomes, and adding glucose oxidase (GOx, G) and ferric ammonium (FAc, F) to form a laser controlled nanosystem (FG@PEL) that inhibits tumour progression under laser irradiation. (**B**) PEVs combined with immunotherapy. Combining Vadimezan with aPDL1-bound platelets (P@aPDL1), in which Vadimezan induces apoptosis of tumour vascular endothelial cells and leads to hemorrhagic necrosis at the tumour site, promotes the recruitment and activation of aPDL1-bound platelets, and produces aPDL1-modified PMPs, which diffuse in the tumour triggering an immune response and causing an anti-tumour effect. (**C**) PEVs combined with chemotherapeutic drugs. Cryopreserved DOX-loaded PLT can reach the tumour microenvironment, where they are activated and release DOX and PEV-DOX under the influence of procoagulant factors and cancer cell-derived extracellular vesicles expressing tissue factor (TF-EV) released by cancer cells, inducing cancer cell death

Given the pivotal role of platelets in tumourigenesis, antiplatelet therapy has emerged as a promising avenue for cancer treatment [181]. Recent studies have highlighted the potential of PEVs as key players in anti-tumour therapy. However, some studies have indicated that certain natural PEVs can potentially affect tumour growth, such as PEVs that inhibit mitochondrial mt-ND2 and *Snora75* (a non-coding small nucleolar RNA) by transferring *miR-24*, resulting in mitochondrial dysfunction and tumour cell growth inhibition [134]. Michael et al. [134] infused human PMPs into tumor allograft model mice and observed significant inhibition of tumour growth. Further studies demonstrated that *miR-24* played a partial role in the effect of PMPs, a finding that was confirmed by in vitro experiments. Furthermore, mice deficient in the thrombin receptor, PAR4, which has been demonstrated to be a primary stimulant of PMP production, exhibit diminished PMP concentrations and accelerated growth of solid tumours. Conversely, PMP infusion impedes tumour growth. These findings offer a potential avenue for targeted tumour therapy. Nonetheless, most researchers have focused on the potential of engineered PEVs in anti-tumour therapy. Studies have demonstrated that engineered PEVs play a pivotal role in anti-tumour therapy when used in conjunction with photothermal therapy (PTT), immunotherapy, and chemotherapy (Fig. 5).

#### Application of engineered PEVs in conjunction with PTT in anti-tumour therapy

The combination of engineered PEVs and PTT has been demonstrated to be beneficial in the treatment of tumours. Ma et al. [182] combined platelet exosomes with photothermal-sensitive liposomes, and added glucose oxidase (GOx, G) and ferric ammonium (FAc, F) to form a laser-controlled nanosystem (FG@PEL). FG@PEL achieved excellent tumour inhibition owing to various synergistic strategies to amplify its therapeutic effect. Wu et al. [183] proposed a local chemical PTT strategy for orthotopic mouse liver cancer promoted by platelet membranes. Polypyrrole (PPy) nanoparticles act as photothermal agents and are encapsulated in the platelet membrane (PLT-PPy-DOX) together with the antitumour drug DOX. These nanoparticles demonstrated excellent antitumour efficiency by inhibiting primary tumour growth and tumour metastasis. Jing et al. [184] encapsulated melanin nanoparticles (MNPs) and DOX in RGD peptide (c(RGDyC))-modified nanoscale platelet vesicles (RGD-NPVs), resulting in RGD-NPVs@MNPs/DOX. Their results demonstrated that RGD-NPVs@MNPs/DOX evaded immune clearance and achieved chemical photothermal elimination of drug-resistant breast tumour cells and the tumour vascular system, resulting in various effects, including reversing multidrug resistance to effectively kill drug-resistant cancer cells; eliminating the tumour vascular system to block the supply of nutrients to the tumour and metastasis; and permanently inhibiting the expression of VEGF, MMP-2, and MMP-9. Chen et al. [185] prepared mesoporous silica-coated bismuth nanorods (BMSNR) disguised using a platelet membrane, resulting in BMSNR@PM. This hybrid material combined PTT and radiotherapy to kill cancer cells in vitro. It has tumour-targeting, immune escape, and radiation sensitisation functions that collectively enable personalised PTT. Ye et al. [186] modified PM on the surface of nanoparticles, in which the chemotherapeutic drug DOX and the photothermal agent indocyanine green were co-encapsulated. In three xenograft and orthotopic breast-tumour-bearing mouse models, the primary tumour was completely ablated, and BC metastasis was effectively inhibited.

#### Application of engineered PEVs in conjunction with immunotherapy in anti-tumour therapy

Combining PEVs with anti-PD1 (aPD1) and anti-PDL1 (aPDL1) antibodies is a promising approach in tumour immunotherapy. Li et al. [26] recently reported the combined effects of vadimezan and aPDL1-bound platelets (P@aPDL1). Vadimezan was found to induce the apoptosis of tumour vascular ECs, leading to haemorrhagic necrosis at the tumour site; promote the recruitment and activation of aPDL1-bound platelets; and produce aPDL1-modified PMPs. The diffusion of particles within the tumour triggered an immune response and exerted antitumour effects. Furthermore, Hu et al. [187] demonstrated that the release of aPDL1 was accelerated following the thrombin-induced activation of platelets and the formation of PMPs. PMP-aPDL1 bound to melanoma cells, resulting in the relocation of aPDL1 to their surface. Hu et al. [188] combined a platelet-modified anti-PD-1 antibody with hematopoietic stem cells (HSCs) and injected them intravenously into mice with leukaemia. The HSC-platelet-aPD-1 conjugate migrated to the bone marrow. Platelet activation promoted the release of anti-PD-1 antibodies through the production of PMPs. This process has been shown to significantly enhance the anti-leukaemic immune response, increase the number of active T cells, induce the production of cytokines and chemokines, and prolong the survival of mice.

#### Application of engineered PEVs in conjunction with chemotherapeutic drugs in anti-tumour therapy

Several studies have investigated the potential of engineered PEVs combined with chemotherapeutic drugs for anti-tumour therapy. A straightforward and expeditious method for preparing DOX-loaded PMPs (PMPDox) has been reported. The efficacy of PMPDox at targeting leukaemia cells and minimising off-target drug delivery has been validated, and potential therapeutic applications of PMPs as biocompatible drug delivery carriers against cancer cells have been explored [189]. In a study by Wu et al. [190], cryopreserved DOX-loaded PLT reached the TME through the blood circulation, where they were activated and released DOX and PEV-DOX under the influence of procoagulant factors and cancer cell-derived EVs expressing tissue factor (TF-EV). This process induced cancer cell death. Liu et al. [191] developed a biomimetic nanocarrier based on platelet membranes (denoted as PEOz-liposome-DOX) by fusing platelet membranes with liposomes. The carrier was created by fusing a platelet membrane with traditional liposomes to form a hybrid membrane vesicle (platelets). The pH-sensitive lipid DSPE-PEOz was incorporated into the platelet membrane to accelerate drug release in an acidic environment, and DOX was simultaneously encapsulated in the particles by hydration. This carrier is suitable for tumour-targeted delivery and pH-induced drug release. When loaded with the chemotherapeutic drug DOX, the nano-preparation effectively delivered DOX to the tumour tissue, exhibiting enhanced antitumour efficacy compared with pH-sensitive liposomes and platelet microparticles, while simultaneously avoiding the occurrence of significant adverse reactions. Wang et al. [192] prepared porous nanoparticles loaded with the anticancer drug bufalin (Bu) using chitosan oligosaccharide (CS)-poly(lactic-co-glycolic acid) (PLGA) as a raw material and then coated them with a platelet membrane to form PLTM-CS-pPLGA/Bu nanoparticles. The PLTM-CS-pPLGA/Bu nanoparticles exhibited active targeting capabilities and were effective at inhibiting tumour growth. Wang et al. [193] constructed platelet-membrane-functionalised buffalin-loaded HMnO_2_ nanoparticles (PLTM-HMnO_2_@Bu NPs). These nanoparticles have been demonstrated to effectively inhibit tumour growth and they represent promising drug-delivery systems for magnetic resonance imaging monitoring and enhanced tumour-targeted therapy. In a recent study, Mei et al. developed a biomimetic platelet-membrane-cloaked paclitaxel (PT) nanocrystal system that can deliver high doses of chemotherapeutic drugs and target coagulation sites caused by surgery or vascular disruptors. This approach enhances antitumour effects and reduces systemic toxicity [194]. Bang et al. [195] designed paclitaxel-loaded nanostructured lipid carriers (PT-NLC) and PLT membrane proteins. They successfully prepared spherical PT-NLC and platelet membrane-coated PT-NLC with affinity and targeting abilities toward tumour cells.

#### Application of engineered PEVs in conjunction with other pharmaceutical agents in anti-tumour therapy

Engineered PEVs have been combined with Western medicine to treat tumours. Zuo et al. [196] used a platelet membrane as a nanocarrier to co-load W_18_O_49_ nanoparticles and metformin (PM-W_18_O_49_-Met NPs). PM protected W_18_O_49_ from oxidation and immune escape and increased the accumulation of W_18_O_49_ at the tumour site through passive enhanced permeability and retention effects and the active adhesion between platelets and cancer cells. Metformin is a typical antidiabetic drug that can alleviate tumour hypoxia by reducing oxygen consumption. Therefore, PM-W18O49-Met NPs have been demonstrated to inhibit tumour growth and induce tumour cell apoptosis. Furthermore, engineered PEVs can be combined with traditional Chinese medicines to treat tumours. Kim et al. [197] designed RBCs and platelet-membrane-coated gold nanostars containing curcumin (R/P-cGNS) and showed that R/P-cGNS can target melanoma cells and avoid macrophage phagocytosis, while inhibiting the inflammatory response.

## Conclusion and future perspectives

Platelets are important components of the TME [9, 74], and thus, they have received increasing attention in recent years. EVs are considered important ‘bridges’ for cell-to-cell information exchange in the TME, mainly because they contain biologically active components (including RNA and proteins). Several studies have identified the potential of PEVs as novel tumour markers and targets for tumour therapy. However, they still have a long way to go, from basic research to clinical translational applications. Moreover, establishing a suitable and reproducible method for producing PEVs with optimal properties remains elusive. Although Aatonen et al. [17] found that stimulation with Ca^2+^ ion carriers produced more PEVs than stimulation with thrombin, collagen, or ADP, proteomic analyses showed that Ca^2+^-generated vesicles carried fewer target proteins. Furthermore, the large-scale production of clinical-grade PEVs is limited by their high cost. Ethical issues may need to be considered if specialised platelet collection procedures are required to provide sufficient platelet concentrates as a source material for PEVs [198]. In addition, there are deficiencies in the purification and characterisation of PEVs. There are several challenges in the purification of PEVs, such as low separation efficiency, sample loss, low recovery and purity of the EVs, and batch-to-batch variations. Therefore, it is vital to provide a comprehensive characterisation of PEVs, encompassing aspects such as size, morphology, concentration, the presence of markers or contents, and the removal of contaminants. There are no standard procedures for the purification and characterisation of PEVs. Furthermore, it is difficult to preserve PEVs after purification. The preservative reagents must have an osmotic pressure similar to that of body fluids to maintain the membrane structure of the phospholipid bilayers; otherwise, the proteins and RNAs with biological functions within the PEVs will be easily inactivated. Moreover, it is difficult to select appropriate PEV blood concentrations as treatment standards because of the lack of standardised testing methods to accurately define PEV concentrations. Engineered PEVs need to be improved to maintain desirable physicochemical properties of the vesicles and increase the loading efficiency. Moreover, most methods used for extracellular vesicle engineering struggle to find a balance between stabilising the load, surface modifications, and maintaining vesicle biocompatibility. Furthermore, materials added to PEVs used as drug delivery vehicles may have potential safety concerns. For example, cationic/ionizable lipids may activate the Toll-like receptor pathway, causing cytotoxicity and leading to inflammation. In addition, PEVs have limitations, such as low targeting efficiency, susceptibility to phagocytosis by the immune system, and difficulty in controlling the dose used.

Although research on PEVs is challenging, future research remains promising. The experience gained from the preclinical and clinical evaluations of PEVs will provide essential information on their quality and safety, including the effective therapeutic dose, their pharmacodynamic and pharmacokinetic properties, and their therapeutic value [198]. This will facilitate the introduction of PEVs in clinics and benefit EV researchers. Although PEVs have been reported to play an important role in tumour immunotherapy, they are mainly in the animal experimentation stage, and it takes a long time from animal experimentation to clinical therapeutic application. Moreover, given that the combination of platelets and AI can be used for early diagnosis, monitoring disease progression, and prognostic assessments of a wide range of tumours, the development of novel detection markers by combining PEVs with AI could be a future trend. Drugs that target platelets may also affect EV secretion, thereby affecting tumour progression. Cilostazol affects phosphodiesterase to inhibit platelet activity and thus, tumour progression [199]; however, whether cilostazol also affects platelet EVs remains unknown. Aspirin administration induces the suppression of plasma PEV cargo protein levels [200], which may explain why aspirin has been shown to inhibit cancer progression. However, aspirin has side effects, such as an increased risk of bleeding, menorrhagia, nausea, and vomiting [201]. Novel drugs that inhibit the release of PEVs induced by cancer cells have promising applications [202], as they are specifically targeted towards PEVs associated with tumour metastasis and do not affect coagulation as they do not inhibit whole-platelet function, thus avoiding several side effects. From a laboratory perspective, the selective elimination of PMPs, but not MPs, from other cell sources has been achieved in the TMEM16F-knockout mouse model [203–205]. Furthermore, inhibiting the ability of tumour cells to take up PEVs through gene therapy is a future cancer treatment strategy.

Because the production of PEVs is highly dependent on parental cell platelets, the development of stem-cell-differentiation-based in vitro platelet generation technologies for the large-scale production of clinical-grade PEVs may be a future direction. In addition, synthetic biology strategies can be used to modify megakaryocytes or platelets by introducing genes of interest to confer additional functions to PEVs [103]. Similar to EVs, PEVs can be designed to deliver suicide gene mRNAs and proteins to target tissues, thereby establishing a novel cancer therapeutic strategy [206, 207]. With the development of high-throughput technology and the increased prevalence of precision therapy research, we believe that, in the near future, the mechanism of PEVs in tumourigenesis and tumour progression will be further elucidated and more breakthroughs in clinical translational applications will be achieved.

## Data Availability

No datasets were generated or analysed during the current study.

## Abbreviations

ACSL: Long-chain fatty acyl-CoA ligase
aPD1: Anti-PD1
aPDL1: Anti-PDL1
AUC: Area under the curve
BC: Breast cancer
CircRNAs: Circular RNA
CLL: Chronic lymphocytic leukemia
CTCs: Circulating tumor cells
DOX: Doxorubicin
ECM: Extracellular matrix
ECs: Endothelial cells
ELISA: Enzyme-linked immunosorbent assay
EPE: Extrapancreatic extension
EVs: Extracellular vesicles
FASN: Fatty acid synthase
HMGB1: High-mobility group box-1
HUVECs: Human umbilical vein endothelial cells
IL-6: Interleukin-6
ILVs: Intraluminal vesicles
LncRNAs: Long non-coding RNAs
MiRNAs: MicroRNAs
MMP: Matrix metalloproteinase
mtDNA: Mitochondrial DNA
MVBs: Multivesicular bodies
MVs: Microvesicles
NCCD: Nomenclature Committee for Cell Death
NPC: Nasopharyngeal carcinoma
NSCLC: Non-small cell lung cancer
OS: Overall survival
OSCC: Oral squamous cell carcinoma
PAI-1: Plasminogen activator inhibitor-1
PDAc-MPs: Platelet-derived activated microparticles
PDAC: Pancreatic ductal adenocarcinoma
PDAp-MPs: Platelet-derived apoptotic microparticles
PDH: Pyruvate dehydrogenase
PEVs: Platelet-derived extracellular vesicles
PiRNAs: PIWI-interacting RNAs
PMPs: Platelet-derived microparticles
PMVs: Platelet-derived microvesicles
PTT: Photothermal therapy
ROC: Receiver operating characteristic
SiRNAs: Small interfering RNAs
SncRNAs: Small non-coding RNAs
TEPs: Tumor-educated platelets
TME: Tumour microenvironment
VEGF: Vascular endothelial growth factor
